# Are Intoxicated Trauma Patients at an Increased Risk for Intraoperative Anesthetic Complications? A Retrospective Study

**DOI:** 10.1155/2020/2157295

**Published:** 2020-03-01

**Authors:** Brian D. Wolf, Swapna Munnangi, Raymond Pesso, Charles McCahery, Madhu Oad

**Affiliations:** ^1^Resident of the Department of Oral and Maxillofacial Surgery, Nassau University Medical Center, East Meadow, New York, USA; ^2^Division of Trauma, Department of Surgery, Nassau University Medical Center, East Meadow, New York, USA; ^3^Department of Anesthesia, Nassau University Medical Center, East Meadow, New York, USA; ^4^American University of the Caribbean School of Medicine, Sint Maarten, Netherlands

## Abstract

**Background:**

The purpose of this study was to correlate intraoperative anesthetic complications of trauma patients with their respective urine toxicology results.

**Methods:**

This retrospective, single-center cohort study at a Level 1 trauma center included patients with the following criteria: (1) trauma admission between January 1, 2010, and December 31, 2016, (2) required surgical intervention, (3) are age 18 and older, and (4) urine toxicology screening was completed. Anesthetic records were evaluated for intraoperative complications.

**Results:**

The final analysis included 847 patients. The mean anesthesia time, American Society of Anesthesiologists physical status classification scores, change in body temperature, anesthetic complication rate, and mortality were not significantly different between urine toxicology positive and negative patients. Of note, a significantly lower proportion of the urine toxicology positive patients were extubated postoperatively in comparison to urine toxicology negative patients (57.32% vs 63.83%).

**Conclusions:**

Trauma patients who presented with a positive urine toxicology screening are not at an increased risk for intraoperative anesthetic complications compared to those with a negative urine toxicology screening. However, our results indicated that the need for postoperative mechanical ventilation increased in the acutely intoxicated trauma patients when compared to those without preinjury intoxication.

## 1. Introduction

The recreational use of controlled substances and illicit drugs has dramatically increased since the turn of the century. Studies estimate an increase of nearly 13% in the past two decades [1–3]. While opioid usage is well publicized, other drugs such as phencyclidine (PCP), methamphetamines, benzodiazepines, barbiturates, and cocaine are also widely abused, frequently with multiple drugs being used together [1]. Trauma patients, when compared to nontrauma patients, have a higher rate of substance abuse. Anywhere from 40 to 80 percent of all trauma patients test positive for illicit drugs upon hospital admission. Drug use can increase the risk of potentially life-threatening complications during the medical management of these patients. As a result of this, accredited trauma centers in the United States use urine toxicology (U-TOX) analysis to actively screen all trauma patients [4].

Drug use has been shown to affect the hospital management of trauma patients [3, 5]. Patients under the influence of drugs may suffer from withdrawal symptoms, require increased dosages of medications to attain adequate sedation and anesthesia, or have impaired drug metabolism [5]. Acutely intoxicated patients run the risk of overdosing if accompanying intoxicants are not accounted for, while chronic abusers may require larger dosages to reach ideal anesthesia due to altered physiology and increased tolerances [6, 7]. Prior research has shown that intoxicated patients may require longer length of stays in the hospital, increased ICU admissions, and the use of mechanical ventilation at increased rates compared to U-TOX negative patients [5]. Contrastingly, there is a growing body of evidence suggesting intoxicated patients do not require altered medical management [5, 8–10].

Anesthesiologists are frequently responsible for the urgent management of trauma patients at all levels of intoxication. Delaying treatment is a common practice for patients who test positive for an intoxicant, but do not require urgent care [11]. The purpose of this study was to compare the association of U-TOX screening results with intraoperative anesthetic complications of trauma patients. Our hypothesis was that U-TOX positive patients would have an increased rate of intraoperative anesthetic complications compared to U-TOX negative patients. We also investigated other perioperative risk factors and their association with U-TOX screening.

## 2. Materials and Methods

The study protocol was reviewed and approved by the Institutional Review Board at the Nassau University Medical Center. This single-center retrospective observational study included trauma patients who were above the age of 18 and admitted to this Level I trauma center between January 1, 2010, and December 31, 2016. The institutional trauma registry and electronic medical records were utilized to obtain data for all trauma patients during this time. The initial sample size included 2066 patients. The study sample was restricted to those that underwent a U-TOX screen upon admission, reducing the sample size to 898 patients. The U-TOX screen was tested for the presence of PCP, cocaine, ethanol, cannabinoids, benzodiazepines, opiates, methamphetamines, and barbiturates with a binary result of positive or negative. Patients with incomplete medical records were excluded from the study, reducing our sample size to 847 patients.

The variables collected included age, gender, body mass index (BMI), anesthesia type, American Society of Anesthesiologists (ASA) physical status classification, anesthetic start and end times, initial and final temperatures, complications noted intraoperatively (defined complication occurring in the time from incision to closure), medications used (Table 1), injury severity score (ISS), and electrolyte levels. Intraoperative complications were interpreted from the anesthetic record. These include tachycardia (independent of incision time, intubation, or extubation), physical injury on intubation, bradycardia, hypoxia, hypertension, hypotension, hyperthermia, hypothermia, emesis, ventricular tachycardia, ventricular fibrillation, pulseless electrical activity, and death. The anesthetic record further provided information on the number of patients who were intubated and those that were extubated immediately following surgery versus those that remained intubated. For patients who were not immediately extubated, medical records were reviewed to estimate length of postoperative intubation. The primary outcome of interest was intraoperative anesthetic complications. Based on the U-TOX screen results, the study cohort was stratified into U-TOX positive and U-TOX negative groups consisting of 328 and 519 patients, respectively.

Statistical analysis was performed using SAS version 9.4 (SAS Institute, Cary, NC). Continuous variables were presented as mean ± standard deviation (SD). Frequency and percentages were used to present categorical variables. Differences in continuous variables were examined using Student's *t* test or the Mann-Whitney *U* test as per the distribution. Pearson's chi-square test or Fisher's exact test was used to examine the association of categorical variables with U-TOX categories. Univariate and adjusted multivariable logistic regression analysis was used to examine factors associated with anesthetic complications. A *p* value <0.05 was considered statistically significant.

## 3. Results

A total of 847 trauma patients met the inclusion criteria. Characteristics of the study sample with subgroups stratified by U-TOX screening results are presented in Table 1. U-TOX screening results were positive for 38.72% of the patients. The mean age of the patients was 51.2 ± 23.5 years. The majority of these patients were male (66.71%) and had a mean ISS of 13.8 ± 10.4. Mean hospital length of stay for these patients was 10.4 ± 14.4 days. Patients with a positive U-TOX result were significantly younger (43.8 ± 20.6 vs 56.0 ± 24.0; *p* < 0.0001) and predominantly male (75% vs 61.46%; *p* < 0.001) and had higher ISS (15.2 ± 11.1 vs 12.9 ± 9.9; *p* < 0.0001), elevated heart rate (93.4 ± 21.6 vs 87.5 ± 18.5; *p* < 0.0001), lower SBP (129.3 ± 30.2 vs 139.4 ± 30.2; *p* < 0.0001), lower GCS (13.1 ± 3.7 vs 14.1 ± 2.7), and lower Cr, BUN, and Glu concentrations relative to those patients with a negative U-TOX result (Table 2).

Anesthesia management-related variables are presented in Table 3. General anesthesia was the most commonly used anesthesia type (85.2%). Overall, the anesthesia complication rate was 9.85%. Paralytics were used in 83.43% of the patients, and cistracurium (44.8%) was the most commonly used paralytic. The majority (93.1%) of the patients were sedated using fentanyl. The mean anesthesia time was 3.2 ± 2.0 hours, and the mean change in body temperature was 0.1 ± 1.4 F. 88.08% of patients were intubated, of which 64.23% of the patients were extubated immediately following surgery and 23.72% had a delayed extubation. The mortality of the study sample was 7.01%. The mean anesthesia time, ASA scores, change in body temperature, anesthetic complication rate, and mortality were not significantly different between U-TOX positive and negative patients. Paralytics (86.89% vs 81.24%) were more frequently used and reversal agents (42.07% vs 52.22%) were less frequently used in U-TOX positive patients relative to U-TOX negative patients. A significantly lower proportion of the U-TOX positive patients were extubated immediately following surgery in comparison to U-TOX negative patients (60.51% vs 64.02%). A significantly higher proportion of U-TOX positive patients had a delayed extubation compared to U-TOX negative patients (30.89% to 19.29%). For patients who were not immediately extubated, the average time to extubation was approximately 47:37:06 (hours:minutes:seconds) with no statistical difference noted between U-TOX positive and U-TOX negative patients (Table 3). In the patients with a positive U-TOX result, ethanol was the most common substance found (26.8%) followed by opiates (15.35%) (Table 4). Univariate logistic regression analysis of factors associated with anesthetic complications is presented in Table 5. A positive U-TOX screen was not independently associated with anesthetic complication. After adjusting for confounding factors, the immediate postoperative extubation status was significantly associated with anesthetic complication. Patients extubated immediately following surgery had 47.6% lower risk of anesthetic complications (Table 6).

## 4. Discussion

The link between trauma patients and acute drug intoxication has been thoroughly documented by trauma centers worldwide, with 40 to 80% of all trauma patients screening positive for one or more intoxicants [1, 4, 5, 8, 12, 13]. These substances, whether ingested acutely or chronically, alter ordinary physiology, increasing the risk for adverse effects and complications during the patient's hospital stay [6, 7]. Surgery must often be postponed for acutely intoxicated patients because of the potential anesthetic risks [10, 11]. However, many trauma patients require urgent treatment forcing anesthesiologists to manage the acutely intoxicated patient. The purpose of this study was to compare intraoperative anesthetic complications of U-TOX positive trauma patients to U-TOX negative trauma patients. Our hypothesis was that U-TOX positive patient would have an increased rate of anesthetic complications compared to U-TOX negative patients.

At this Level 1 trauma center, trauma patients were positively screened 38.72% of the time. These U-TOX positive patients were usually male (75%) and usually younger (43.8 years old) when compared to the U-TOX negative population. These results are comparable with the demographic profile noted in past studies [1, 4, 5, 8, 12]. The most common drugs found in the screening were ethanol (26.80%) and opiates (15.35%) followed by cannabis and benzodiazepines (7.56% and 6.8%, respectively). This is consistent with the substantial rise in prescription drug abuse noted in the past two decades [1, 2, 6, 13]. No significant changes in the medications used for the anesthetic management of U-TOX positive and U-TOX negative patients were found in our study. Fentanyl and cisatracurium were the most commonly used sedative and paralytic medications used, respectively. Furthermore, no differences were noted in mean anesthesia time, changes in body temperature, intraoperative complication rates, or mortality between the U-TOX positive and U-TOX negative patients. Interestingly, U-TOX positive trauma patients were significantly less likely to be extubated immediately following surgery compared to U-TOX negative trauma patients (60.51% vs 64.02%). U-TOX positive patients also had a significantly higher risk of delayed extubation compared to U-TOX negative patients (30.89% to 19.29%). However, both U-TOX positive and negative patients had similar lengths of prolonged intubation postoperatively (42 : 35 : 14 to 52 : 27 : 49).

These results appear conflicting but are consistent with the current body of the literature. Multiple studies have found that trauma patients with positive screening for intoxicants are more likely to require mechanical ventilation following admission [3, 5, 14, 15]. Yet, there is little other evidence that alterations to the medical and anesthetic management of these patients are needed as their outcomes and complication rates are comparable to patients who tested negative for intoxicants [1, 5, 9, 10, 14]. Ryb and Cooper found that the outcomes of trauma patients who required surgery within 24 hours of admission were not negatively affected by a positive cocaine screening [10]. Rootman et al. found that drug intoxication of trauma patients did complicate their admission, but did not increase overall length of stay, ICU admission, or mortality [14].

This study has several limitations. The study is prone to bias inherent to retrospective studies. This includes missing information and poor information quality. For instance, the time of delayed extubation was the length of time that elapsed between the end of surgery and the first note in the medical chart confirming the patient was extubated, not necessarily the exact time of extubation. Furthermore, data points could not be used for patients who underwent supplemental procedures (tracheostomy), patient mortality, and patients who were transferred to another facility postoperatively. There may also be breaks in protocol where some trauma patients were not screened or results were never submitted. It also cannot take into account the variability among anesthetic management and techniques. This includes drug shortages over the course of the study and changes in hospital protocol or staffing that may lead to different medications being used.

Another major limitation of our study is that we assume a positive U-TOX screen implies acute patient intoxication. Despite the ubiquitous use of these screening assays in patient care, the recent literature suggests that these screenings can result in “false positives” and “false negatives,” leading to misinterpretation and mismanagement by practicing physicians [16, 17]. It is important to remember that acute intoxication is a clinical diagnosis, requiring information from the patient's physical examination and history. Future studies should include patient's past medical history and physical examination to control for those who are chronic users of the drugs they are being screened for and not acutely intoxicated.

## 5. Conclusion

We believe this is the first study to investigate intraoperative anesthetic complications in U-TOX positive trauma patients. Many studies delineated medical requirements and surgical complications of patients being cared for by the surgical and medical teams following a trauma, but few go into the anesthetic management of such patients [1, 3, 5–7, 9–12, 14, 15]. Similar to multiple surgical and medical studies, our results found that postoperative mechanical ventilation is often needed for these patients when compared to those without preinjury intoxication [3, 5, 14, 15]. Further studies are needed to clarify how preinjury intoxication affects ventilation following a trauma and on the anesthetic management of acutely intoxicated patients as a whole. In summary, this study found that trauma patients who presented with a positive drug screening are not at an increased risk for intraoperative complications but revealed an increased risk of delayed extubation, requiring mechanical ventilation postoperatively.

## Figures and Tables

**Table 1:** Medications used.

Paralytics	None, succinylcholine, cisatracurium, rocuronium, vecuronium, others
Sedatives	None, fentanyl, propofol, ketamine, hydromorphone, midazolam, etomidate, morphine, remifentanil
Antiemetics	None, dexamethasone, ondansetron, prochlorperazine, diphenhydramine, metoclopramide, scopolamine, others
Reversal agents	None, naloxone, flumazenil, sugammadex, neostigmine
Beta blockers	Esmolol, labetalol, metoprolol
Antihypertensives	Hydralazine, nicardipine
Vasopressors	Phenylephrine, epinephrine, ephedrine, norepinephrine, vasopressin
Others	Atropine, amiodarone, potassium, calcium, bicarbonate, albuterol, magnesium

**Table 2:** Demographic and clinical characteristics of the study sample.

Variable	Total sample (*n* = 847)	Urine toxicology positive (*n* = 328)	Urine toxicology negative (*n* = 519)	*p* value
Age	51.2 ± 23.5	43.8 ± 20.6	56.0 ± 24.0	<0.001^*∗*^
Sex				
Female	282 (33.3%)	82 (25.0%)	200 (38.54%)	<0.001^*∗*^
Male	565 (66.71%)	246 (75.0%)	319 (61.46%)	
Body mass index	26.8 ± 6.0	27.0 ± 6.1	26.7 ± 5.9	0.6000
Hospital length of stay (days)	10.4 ± 14.4	10.9 ± 14.7	10.1 ± 14.3	0.3860
Injury severity score	13.8 ± 10.4	15.2 ± 11.1	12.9 ± 9.9	0.0026^*∗*^
Revised trauma score	7.4 ± 1.2	7.3 ± 1.4	7.6 ± 1.0	0.0008^*∗*^
Probability of survival	0.9 ± 0.2	0.9 ± 0.2	0.9 ± 0.2	0.1122
Systolic blood pressure	135.5 ± 30.6	129.3 ± 30.2	139.4 ± 30.2	<0.001^*∗*^
Diastolic blood pressure	79.5 ± 19.2	78.0 ± 19.7	80.5 ± 18.9	0.0700
Heart rate	89.8 ± 20.0	93.4 ± 21.6	87.5 ± 18.5	<0.001^*∗*^
Glasgow coma scale	13.8 ± 3.2	13.1 ± 3.7	14.1 ± 2.7	<0.001^*∗*^
Sodium	144.9 ± 129	140.7 ± 3.2	142.6 ± 55.7	0.4440
Potassium	4.4 ± 10.5	4.9 ± 16.8	4.1 ± 0.6	0.4330
Chloride	105.9 ± 31.4	105.6 ± 4.5	106.2 ± 40.0	0.7100
Creatinine	24.5 ± 7.8	23.4 ± 3.8	25.2 ± 9.5	<0.001^*∗*^
Blood urea nitrogen	18.1 ± 10.2	15.1 ± 7.4	20.0 ± 11.2	<0.001^*∗*^
Glucose	137.8 ± 55.6	132.9 ± 45.4	140.9 ± 61.1	0.0305^*∗*^

Values are expressed as mean ± sd or *n* (%). ^*∗*^*p* < 0.05.

**Table 3:** Anesthetic management.

Variable	Total sample (*n* = 847)	Urine toxicology positive (*n* = 328)	Urine toxicology negative (*n* = 519)	*p* value
Anesthesia type				0.0128^*∗*^
General	722 (85.24%)	289 (88.11%)	433 (83.43%)	
IVS	16 (1.89%)	8 (2.44%)	8 (1.54%)	
Local/regional	56 (6.61%)	10 (3.05%)	46 (8.86%)	
General and IVS	3 (0.35%)	1 (0.30%)	2 (0.39%)	
General and local/regional	22 (2.60%)	11 (3.35%)	11 (2.12%)	
IVS and local/regional	28 (3.31%)	9 (2.74%)	19 (3.66%)	
Anesthesia time (hrs)	3.2 ± 2.0	3.3 ± 2.1	3.2 ± 1.9	0.7850
Change in temperature (F)	0.1 ± 1.4	0.0 ± 1.3	0.1 ± 1.4	0.6725
ASA^a^				0.0534
1	50 (5.95%)	20 (6.13%)	30 (5.83%)	
1E	62 (7.37%)	27 (8.28%)	35 (6.80%)	
2	186 (22.12%)	73 (22.39%)	113 (21.94%)	
2E	142 (16.88%)	64 (19.63%)	78 (15.15%)	
3	181 (21.52%)	53 (16.26%)	128 (24.85%)	
3E	70 (8.32%)	24 (7.36%)	46 (8.93%)	
4	61 (7.25%)	20 (6.13%)	41 (7.96%)	
4E	66 (7.85%)	34 (10.43%)	32 (6.21%)	
5E	21 (2.50%)	10 (3.07%)	11 (2.14%)	
E	2 (0.24%)	1 (0.31%)	1 (0.19%)	
Anesthesia complications^b^	83 (9.85%)	37 (11.28%)	46 (8.93%)	0.2645
Paralytics^c^	705 (83.43%)	285 (86.89%)	420 (81.24%)	0.0313^*∗*^
Sedatives^c^	833 (98.58%)	324 (98.78%)	509 (98.45%)	0.6946
Antiemetics	526 (62.10%)	193 (58.84%)	333 (64.16%)	0.1200
Reversal agents^c^	408 (48.28%)	138 (42.07%)	270 (52.22%)	0.0040^*∗*^
Other drugs	644 (76.21%)	239 (72.87%)	405 (78.34%)	0.0687
Intubated	724 (88.08%)	288 (91.72%)	436 (85.83%)	0.0130^*∗*^
Immediate extubation	528 (64.23%)	190 (60.51%)	338 (64.02%)	0.0800
Delayed extubation^d^	195 (23.72%)	97 (30.89%)	98 (19.29%)	<0.001^*∗*^
Time until extubation (hr:min:sec)^e^	47:37:06	42:35:12	52:27:49	0.3909
Mortality^f^	59 (7.01%)	26 (7.95%)	33 (6.41%)	0.3925

Values are expressed as mean ± sd or *n* (%). ^*∗*^*p* < 0.05. IVS, intravenous sedation; ASA, American Society of Anesthesiologists physical status classification; E, emergency; ^a^missing *n* = 6; ^b^missing *n* = 4; ^c^missing *n* = 2; ^d^missing *n* = 25; ^e^missing *n* = 115 due to hospital transfers, patient mortality, patients receiving tracheostomy postoperatively, or missing information; ^f^missing *n* = 5.

**Table 4:** Urine toxicology screening positive drugs found.

Drug found	n (%)
Ethanol	227 (26.80%)
Amphetamine	1 (0.12%)
Barbiturates	2 (0.24%)
Benzodiazepine	58 (6.8%)
Cannabis	64 (7.56%)
Methadone	25 (2.95%)
Cocaine	33 (3.90%)
Opiates	130 (15.35%)
Phencyclidine	2 (0.24%)

**Table 5:** Unadjusted logistic regression analysis of factors associated with intraoperative anesthetic complications.

	Odds ratio	95% confidence interval	*p* value
Age	1.004	0.995–1.014	0.3595
Urine toxicology screen positive (reference: urine toxicology screen negative)	1.296	0.821–2.047	0.2654
Male (reference: female)	0.980	0.607–1.583	0.9347
Injury severity score	1.046	1.028–1.064	<0.001^*∗*^
Revised trauma score	0.758	0.659–0.871	<0.001^*∗*^
Glasgow coma scale	0.899	0.851–0.948	<0.001^*∗*^
Ethanol	1.326	0.815–2.158	0.2562
Cocaine	1.131	0.367–3.484	0.8299
Opiates	0.652	0.298–1.425	0.2836
Benzodiazepine	0.904	0.351–2.327	0.8340
Cannabis	0.819	0.318–2.108	0.6782
Methadone	1.714	0.541–5.429	0.3594
Extubated (reference: not extubated)	0.352	0.216–0.573	<0.001^*∗*^

^*∗*^ *p* < 0.05.

**Table 6:** Multivariable logistic regression analysis of factors associated with intraoperative anesthetic complications.

	Odds ratio	95% confidence interval	*p* value
Urine toxicology screen positive (reference: urine toxicology screen negative)	1.175	0.714–1.932	0.5263
Injury severity score	1.023	0.999–1.048	0.0572
Revised trauma score	0.924	0.598–1.427	0.7209
Glasgow coma scale	1.013	0.857–1.197	<0.001^*∗*^
Extubated (reference: not extubated)	0.524	0.283–0.969	0.0393^*∗*^

^*∗*^ *p* < 0.05.

## Data Availability

Deidentified data and material can be provided from the corresponding author upon request.
